# Stepping into the role of Editor‐in‐Chief of the *Journal of Cell Communication and Signaling*

**DOI:** 10.1002/ccs3.70000

**Published:** 2025-02-14

**Authors:** Brahim Chaqour

**Affiliations:** ^1^ University of Pennsylvania Perelman School of Medicine Philadelphia Pennsylvania USA

**Keywords:** cell‐cell communication, cell signaling, editor‐in‐chief, JCCS, scientific publication

## Abstract

I am deeply honored to have been appointed as the new Editor‐in‐Chief (EiC) of the *Journal of Cell Communication and Signaling* (*JCCS*). As I step into this role of EiC, I look forward to continuing collaborating with the dedicated readership, expert reviewers, editorial board members, and the publisher of the journal to advancing knowledge in research on cell communication and signaling governing key biochemical and physiological processes both in normal and pathological conditions. *JCCS* remains dedicated to publishing cutting‐edge research, while continuously expanding the range of submissions and refining the review and editorial processes to ensure a more inclusive and impactful scientific contribution. I would like to thank the founder of *JCCS* and my predecessor EiC, Prof. Bernard Perbal and his right hand Annick Perbal for their enormous contribution to *JCCS*, and their crucial role shaping the Journal into a platform that welcomes innovative research and enhances our understanding of cellular communication networks in health and diseases and for steering us safely over the last two decades through the increasingly challenging scientific publication landscape. In this editorial, I share some of my reflections, ideas, acknowledgments, and aspirations for the future of the journal.


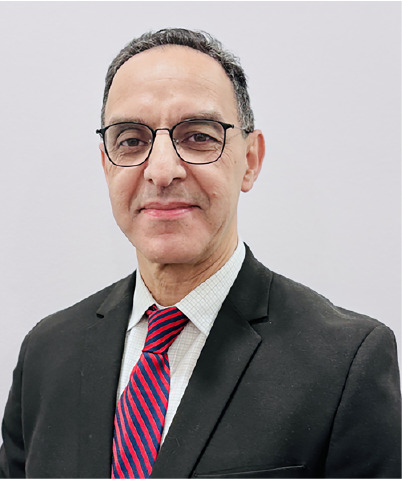


The beginning of 2025 marks almost two decades of publication of the *Journal of Cell Communication and Signaling* (*JCCS*).^1^ As a journal dedicated to publishing novel research findings that decipher the “languages” of all biological systems, we have remained steadfast in our NorthStar mission of publishing and informing the scientific community about the latest discoveries and clinically relevant knowledge and methodologies in the field of cell communication and signaling governing key biochemical and physiological steps, both in normal and in pathological conditions such as fibrosis, cancer and metabolic diseases. A key focus is the impact of intracellular signaling, as well as cell–cell and cell–matrix interactions, not only on the development, function, and malfunction of cells, tissues, and organs but also on potential therapies for a wide range of proliferative, cardiovascular, neurodegenerative, and inflammatory diseases.

We are deeply grateful to our readers, reviewers, editorial board members, and the executive and associate editors of the journal. Their dedication—donating precious time and effort to carefully and thoughtfully review all manuscripts—ensures that we remain relevant and impactful in the ever‐evolving and competitive field of scientific publishing. I also extend my thanks to the hardworking individuals at Wiley, who work behind the scenes to facilitate the administration, manuscript processing, and production of the journal. All of us affiliated with *JCCS* look forward to the next decade of publication and the journal's continued evolution in line with the emerging scientific discoveries that will undoubtedly take place.

2025 also marks the beginning of my tenure as Editor‐in‐Chief (EiC) of *JCCS*. It is with profound gratitude and a deep sense of purpose that I assume this role, having previously served as an editorial board member, Associate Editor, and Executive Editor of the journal. These roles have allowed me to engage in enriching scientific discussions and productive exchanges with authors, co‐authors, editors, and publishers, and this have greatly contributed to my professional development and, I hope, scientific maturity. It also provided me with a unique opportunity to collaborate with some of the most outstanding researchers worldwide in my chosen fields of extracellular matrix (ECM) signaling, mechanosensing pathways, intracellular signalomics, and cell–cell interactomics, particularly in models of vascular and neurovascular system development and diseases. This collaborative research is key to identifying druggable pathways in obstructive, vascular, and neurodegenerative diseases. As a journal editor and reviewer, these experiences have strengthened my passion and resolve to advance both mechanistic/“interpretability” studies in biological models, and applied research aimed at finding immediate solutions and treatments for known pathologies.

My journey on the *JCCS* editorial board has also been profoundly shaped by the skills and dedication of Dr. Bernard Perbal and his soulmate Annick Perbal.^2^ Their leadership and initiative have been an enduring source of inspiration. Dr. Perbal founded *JCCS* as the official journal of the International CCN Society (ICCNS)^3^ to support the society's mission and to increase awareness of the research community of scientific discoveries about the matricellular proteins of the cellular communication network (CCN) gene family. Bernard's pioneering contributions to research on CCN protein structure, signaling, and function in various biological models have significantly advanced our understanding of these ECM molecules, which play a functional rather than structural role in the extracellular environment.^4^ Bernard regularly delivers keynote talks at the ICCNS biannual meetings, constantly appealing to the creativity and innovative thinking of the meeting's attendees to undertake new studies to fill the gaps in our knowledge on CCN protein‐dependent and independent signaling pathways. As a result, an extensive body of work on CCN proteins has been published in *JCCS* and other prestigious journals. Many multi‐omics studies have highlighted the importance of cell communication and signaling pathways in key biological processes, such as regulation of cell growth, differentiation, migration, ECM synthesis, and cellular organelle biogenesis, degradation and function.^5^, ^6^ Naturally, expanding *JCCS*'s scope to include impactful research on all aspects of cell communication and signaling became essential.^7^

As the founding Editor and EiC in 2024, Bernard played a crucial role in shaping *JCCS* into a platform that welcomes innovative research that enhances our understanding of cellular communication in health and disease. Bernard's thoughtfulness, courage, wit, and perseverance will always be remembered by all who have worked with or known him. I also want to acknowledge Dr. Andrew Leask, who served as EiC before the transition of *JCCS* from Springer to Wiley. Dr. Leask significantly contributed to the journal's expansion and success, and the current standing and reputation of *JCCS* are, at least in part, a direct result of his dedication.^1^ I, personally, embark on this new EiC role with deep gratitude for all my predecessors, editorial board members, reviewers, and associate and executive editors, whose selfless commitment has shaped *JCCS*.

It is a privilege to build upon the legacy of my predecessors, and I am committed to carrying forward their values of innovation and excellence. I also recognize that times of transition can be challenging and bring about uncertainties regarding what lies ahead. A significant transitional period for *JCCS* was its shift from a primarily subscription‐based model to a fully open‐access (OA) journal, supported by article processing charges (APC) paid by funding agencies, sponsors, or authors' affiliated institutions.^8^ This transition to OA was largely driven by Plan S, an initiative unifying research funders to ensure open access to research findings for the global community.

Although the APC system might seem like an additional cost for principal investigators, it is a relatively small expense compared to the broader costs of conducting research, such as personnel, supplies, reagents, equipment, animal per diem, facilities etc.^9^ Although this system may also seem financially advantageous to publishers, it also requires a discount and waiver policy for authors in low‐ and middle‐income countries, with policies varying between publishers and journals. It is noteworthy that publishers bear the significant cost of infrastructure required for hosting, reviewing, editing, and formatting journal issues. Interestingly, OA journals have seen rapid growth in recent years. The Directory of Open Access Journals now lists over 21,000 journals, up from 7000 in 2017. Scopus indexes more than 42,000 journals, and a significant share of these are OA. By the end of 2024, the number of OA articles indexed in Scopus surpassed 23 million. In line with these statistics, McKiernan et al. reported that OA papers have a considerably higher citation rates than non‐OA papers, with an increase ranging from 36% to 172%.^8^ As an OA journal, *JCCS* adheres to all criteria for quality, editorial oversight, indexing, and sustainability, ensuring that valuable information remains accessible without compromising principles.

I am excited that, together with our ad hoc reviewers, editors, and associate and executive editors, we will continue to enhance *JCCS*'s standing as a leading global journal in intra‐ and extracellular signaling research in both experimental and translational science. The high impact and prestige of any journal are only possible because of the quality and originality of the scientific publications submitted to and published in it. As a team, we will encourage research that addresses complex systems biology questions that may have been too challenging to tackle in the past. We will ensure that the journal's scope is aligned with the latest research trends and emerging methodologies within the field of cell communication and signaling. Engaging with current scientific debates and high‐impact research areas will attract more and high quality submissions and boost relevance.

We also aim to learn from research across the globe and achieve more by being inclusive and promoting scientifically sound, evidence‐based work from researchers worldwide. Along these lines, we will expand the pool of reviewers to include experts from different geographical regions, institutions, and career stages. This will not only provide a broader perspective on submissions but also improve the credibility and fairness of the review process.

Another step in this direction is to revamp, refresh and diversify the editorial board to include top experts from different subfields and regions. A broader editorial board can provide a wider array of expertise and a better understanding of research trends. We will strongly support the increased involvement of junior researchers in the editorial and review processes, encouraging them to take an active role in the journal's work and use their contributions to shape their careers and the future of *JCCS*. An editorial board that is diverse in terms of geography, ethnicity, and gender is essential to our continued success. Editorial board members and ad hoc reviewers lend their time and talent to the authors and us editors, in arriving at a published article. Therefore, each year, *JCCS* will celebrate the contributions of our editors and ad hoc reviewers by awarding a “Best Editor” and “Best Reviewer” plaque‐award, with the opportunity for awardees to publish an article in *JCCS* free of APC charges.

Finally, we will work with our publisher to further simplify the submission process for authors and to streamline the peer review system for editors and administrators. This reduces barriers and improves the quality and quantity of submissions.

By focusing on these strategies, our team will foster long‐term growth in both readership and citations. To our readers, we welcome your suggestions, ideas, and concerns on how we can better serve the research community.
